# Near-Infrared Imaging With Indocyanine Green for the Treatment of Endometriosis: Results From the Gre-Endo Trial

**DOI:** 10.3389/fonc.2021.737938

**Published:** 2021-11-15

**Authors:** Luigi Carlo Turco, Giuseppe Vizzielli, Virginia Vargiu, Salvatore Gueli Alletti, Maria De Ninno, Gabriella Ferrandina, Luigi Pedone Anchora, Giovanni Scambia, Francesco Cosentino

**Affiliations:** ^1^ Department of Women’s and Children’s Health, Fondazione Policlinico Universitario A. Gemelli IRCCS, Rome, Italy; ^2^ Department of Gynecologic Oncology, Gemelli Molise, Campobasso, Italy; ^3^ Department of Pathology, Gemelli Molise, Campobasso, Italy; ^4^ Università Cattolica del Sacro Cuore, Rome, Italy; ^5^ Department of Medicine and Health Sciences “Vincenzo Tiberio”, Università degli Studi del Molise, Campobasso, Italy

**Keywords:** near-infrared imaging, indocyanine green, deep infiltrating endometriosis, personalized medicine, gynecological surgery

## Abstract

**Introduction:**

A current challenge for endometriosis surgery is to correctly identify the localizations of disease, especially when small or hidden (occult endometriosis), and to exactly define their real extension. The use of near-infrared radiation imaging (NIR) after injection of indocyanine green (ICG) represents one of the most encouraging method. The aim of this study is to assess the diagnostic value of NIR-ICG imaging in the surgical treatment of endometriosis compared with the standard of treatment.

**Material and Methods:**

The Gre-Endo trial is a prospective, single-arm study (NCT03332004). After exploring the operatory field using the white light (WL) mode, patients were injected with ICG and then observed in NIR mode. All suspected areas were classified and chronicled according to lesions visualized only in WL, NIR-ICG, or in the combination of both. Lesion not visualized in WL was considered as suspect occult lesion (s-OcL). In addition, a random control biopsy from an apparent negative peritoneum visualized in WL and NIR-ICG imaging was taken for all patients (control cases). All lesions removed were considered “suspect endometriosis” until pathology.

**Results:**

Fifty-one patients were enrolled between January 2016 and October 2019. A total of 240 suspected lesions have been identified with both methods (WL + NIR-ICG). Two hundred and seven (86.2%) lesions out of the overall 240 were visualized with WL imaging, and 200 were confirmed to be pathologic (true positive for WL). The remaining 33/240 (13.75%) (false negative for WL) lesions were identified only with NIR-ICG imaging and collected as s-OcL. All 33 s-OcLs removed were confirmed to be pathologic (c-OcL = 100%). NIR-ICG vision showed PPV of 98.5%, NPV of 87.1%, Se of 87%, and Sp of 98.5%, confirming that this kind of imaging is an excellent diagnostic and screening test (*p* = 0.001 and *p* = 0.835, according to McNemar’s and Cohen’s kappa tests, respectively).

**Conclusions:**

The use of NIR-ICG vision alone and combined with WL showed good results in intraoperative detection rate and fluorescence-guided surgery of endometriosis. Furthermore, NIR-ICG allowed surgeons to remove occult lesions that otherwise would remain, leading to possible greater postoperative pain and a higher risk of persistence and relapse.

## Introduction

Endometriosis is considered a public health problem compromising the social, employment, financial, and reproductive quality of life of the patients (1). When pharmacologic treatment fails, surgical treatment can improve quality of life and fertility by radically removing extra-ovarian endometriosis localizations using the best minimally invasive techniques such as laparoscopy, the current gold standard of treatment (2–4).

Early technical difficulties have been overcome by surgeon experience and the refinement of techniques; a frequent current challenge involves identifying endometriosis localizations, especially when small or hidden (occult endometriosis) (5–9), to not leave out disease and determine a possible “undertreatment” and/or to predispose patients to possible recurrences.

Indeed, there is evidence that postoperative recurrence of endometriosis may be due to incomplete resection during the primary surgery (8).

Furthermore, eradicating surgery for endometriosis presents the risk of “overtreatment” as well, since the surgeon usually may remove lesions suspected of being endometriosis that are not pathologically confirmed to be endometriosis from 16% to 53% of cases (8). The excessive dissection and resection of heathy tissues surrounding the diseased, moreover, could determine postoperative surgical and functional morbidity (10–13).

Indocyanine green (ICG) is nowadays increasingly used in gynecological surgery, both in oncological and benign fields (14–16). It is frequently used in the identification of lymphatic tissue (17, 18), but if injected intravenously, it binds to plasma proteins and persists in the vascular system, helping in the definition of the vascular network (19).

Given the typical neovascularization of endometriosis, related to chronic inflammation, the visualization of abnormal areas of peritoneal vascularization could be useful to better identify and define the endometriosis lesions in their real extension and to visualize the lesions even when not obvious, as in puckered peritoneal lesions (8–11, 20). For all of this, several methods have been proposed to improve the intraoperative treatment of endometriosis through enhancing the human vision power, with encouraging results (21–23).

The use of cameras with near-infrared radiation imaging (NIR) after injection of ICG represents one of the most encouraging methods in this experimental scenario, demonstrating a good profile of safety and accuracy as an intraoperative diagnostic method (9–11, 24).

The aim of this study is to assess the diagnostic value of NIR-ICG imaging in the surgical treatment of endometriosis compared with the standard of treatment, that is laparoscopy in white light (WL), and the standard diagnostic method, that is pathologic finding.

## Materials and Methods

The Gre-Endo trial is a prospective, single-arm study (ClinicalTrials.gov Identifier: NCT03332004) carried on at the Fondazione Policlinico Universitario “A. Gemelli”—IRCCS, Rome, Italy, and Gemelli-Molise, Campobasso, Italy. The local ethics committee approved the experimentation (Prot.sf. A.287/C.E./2013).

### Materials

The NIR-ICG camera system adopted for the study was the Olympus ICG Imaging System Prototype based on the VISERA Pro System (custom camera head, modified light source, and modified camera control unit; Olympus Europa Holding GmbH, Hamburg, Germany), the merchandized camera head CH-S200-XZ-EB connected to VISERA ELITE II system with NIR filter (Olympus Europa Holding GmbH, Hamburg, Germany), and the IMAGE1 S™ Rubina imaging technology from KARL STORZ.

The ICG adopted for intravenous injection during the procedures was Pulsion (PULSION Medical Systems SE, Feldkirchen, Germany) and VerDye (Diagnostic Green GmbH, Aschheim-Dornach, Germany).

### Patients

Inclusion criteria were suspected endometriosis with surgical indication for treatment needing laparoscopic and pathologic confirmation. Patients were triaged to surgery according with the common indications for endometriosis (25). Exclusion criteria were age <18 and >47 years at the time of surgery. Other exclusion criteria were a history of allergic reactions attributed to compounds of similar chemical or biologic composition to ICG; pregnancy or breastfeeding period; active participation of the patient to other drug, biologic, and/or device study; the presence of medical conditions contraindicating general anesthesia or standard surgical approaches; and any contraindicating medical condition, according to the discretion of the investigator, that made the subject a poor candidate for the investigational procedure.

Patients with ovarian endometriosis and/or endometriosis of the fallopian tubes were excluded from the study because of the intraoperative lack of fluorescence of the ovaries and the diffuse fluorescence of the tubes because of physiological vascular web density at preliminary pilot surgeries.

After obtaining informed consent, patients were included in the study and they could withdraw from the study at any time without impacting treatment. Patient demographic features and preoperative pain were scored using the visual analog scale (VAS) (26), and the intraoperative classification of endometriosis severity scheduled according to the revised American Fertility Society (rAFS) (27). All data were prospectively collected.

### Method and Surgical Procedure

All the procedures were performed by a team of three well-trained surgeons with >10 years of experience in minimally invasive techniques for endometriosis.

**Figure 1:**
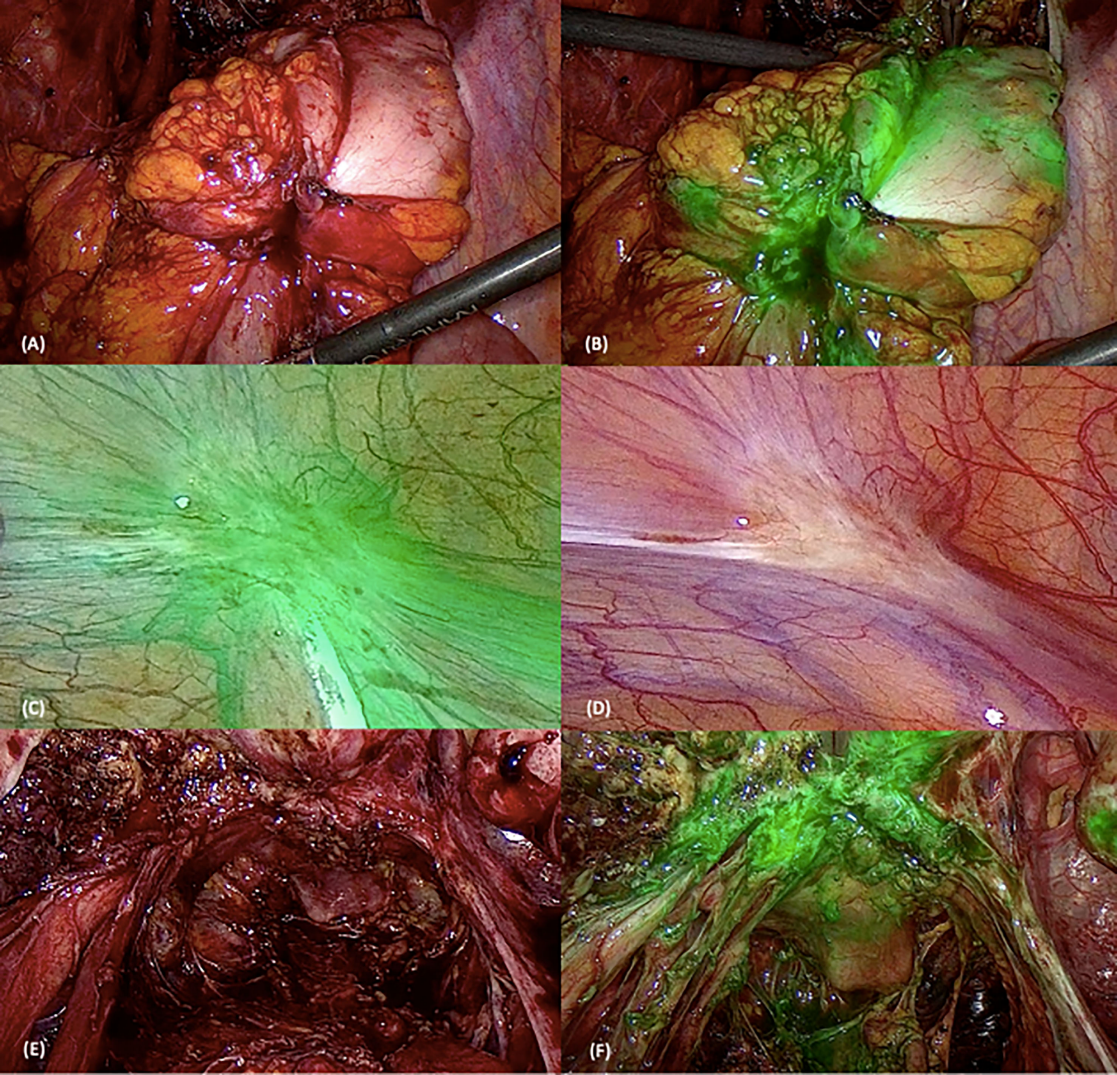
Surgical images using white light (WL) and near-infrared indocyanine green (NIR-ICG) mode. The first two pictures represent a rectosigmoid nodule using WL **(A)** and NIR-ICG mode **(B)**. **(C, D)** Superficial peritoneal lesion in WL **(D)** and NIR-ICG **(C)**. The last two pictures show retrocervical lesions with uterosacral ligament involvement using the two vision systems [WL in **(E)** and NIR-ICG in **(F)**].

During surgery, the abdomen and pelvis were visually inspected using direct laparoscope visualization under WL conditions. The surgeon prepared the operating field by adhesion lysis exposing the *torus uteri* and the *ovarian fossa* and freeing the bowel from eventual retrocervical nodule attachment. All suspected areas were classified as either peritoneal superficial endometriosis (PE) or deep infiltrating endometriosis (DIE). All suspected PE was classified as white, black, and red lesions and documented with their anatomic location in the surgical record under WL condition. Similarly, all suspected DIE lesions were also recorded with their anatomic location in the surgical record (retrocervical, vaginal, rectosigmoid, bladder lesions, etc.) (8) under WL condition. The patient was then administered with 0.25 mg/kg of ICG intravenously. After an interval of time from a minimum of 5 min, NIR-ICG imaging was activated and the whole surgical field inspected with this filter (**Figure 1**). It was necessary to wait to permit blood flow washout of ICG and its accumulation in the third space of neovascularized areas. All suspected lesions for endometriosis (PE and DIE) were tabulated and chronicled according to lesions visualized only in WL, only in NIR-ICG, or in the combination of both. In addition, a random control biopsy from an apparent negative peritoneum visualized in WL and NIR-ICG imaging was taken for all patients (control cases). Every specimen resected during surgery was considered as “suspect lesion” for endometriosis when visualized with WL and/or with NIR-ICG until pathology confirmation. If a suspect lesion had been visualized with NIR-ICG and not in WL or, conversely, only with WL, it was named “suspect occult lesion” (s-OcL), and only after conformation by pathology, it has been considered “confirmed occult lesion” (c-OcL).

All specimens resected were analyzed by a dedicated pathologist that, even when facing with macroscopically negative tissue samples, embedded in paraffin the specimens *in toto* and analyzed them at multiple levels. A surgical specimen was considered as “pathologic” when containing endometriosis *foci* (stroma and/or gland and/or hemosiderin) and/or acute or chronic sclerosing inflammatory infiltrate (28, 29).

Perioperative complications have been reported with the extended Clavien–Dindo classification (30).

### Statistical Analysis

The primary objective of the study was to assess the feasibility of NIR-ICG to identify endometriosis lesions and distinguish the surrounding tissue in comparison with WL. The secondary objective was to assess the power of identifying OcL and the power of the test combining the two methods of visualization (WL plus NIR-ICG).

Normally, WL is the intraoperative gold standard imaging technique for detecting endometriosis, while pathology the definitive confirmation test.

We tested the null hypothesis that the possibility of correctly identifying endometriosis could improve from 85% with WL visualization to 100% when assessed together with NIR-ICG. The sample size was calculated according to the Simon two-stage design (31) using an alpha error of 0.01 and a beta error of 0.90. Considering a patient dropout of approximately 10%, the study was planned to enroll at least 47 women.

Because the control biopsy was achieved from a negative peritoneum using WL and NIR-ICG imaging for all women, the true-negative lesions were defined as the negative lesions for endometriosis that were correctly identified as negative by WL or NIR-ICG imaging; the false-negative lesions were defined as not correctly identified by WL or NIR-ICG imaging. The true-positive lesions were the positive lesions for endometriosis that were correctly identified by WL or NIR-ICG imaging; the false-positive lesions were the lesions identified as positive for endometriosis by WL or NIR-ICG imaging that were not pathologically identified as endometriosis. Sensitivity, specificity, positive predictive value (PPV), negative predictive value (NPV), and overall accuracy were calculated for each visualization. Sensitivity (Se) was defined as the number of positive lesions for endometriosis that were correctly identified (true positives) divided by the total number of positive lesions for endometriosis (true positives + false negatives). Specificity (Sp) was defined as the number of negative lesions for endometriosis that were correctly identified (true negatives) divided by the total number of negative lesions (true negatives + false positives). PPV was calculated as the number of true positives divided by the total number of positive results (true positives + false positives), and NPV was defined as the number of true negatives divided by the total number of negative results (true negatives + false negatives). Accuracy was calculated as the number of true positives plus true negatives (total correct number) divided by the total number of patients studied. Sensitivity, specificity, and accuracy were compared using McNemar’s and Cohen’s kappa tests. The diagnostic performances of WL and NIR-ICG imaging were calculated per patient as well as per lesion. Statistical calculations were performed using the Statistical Package for Social Sciences (Version 17.0; SPSS Inc., Chicago, IL, USA).

The receiver operating characteristic (ROC) curve was designed to assess the diagnostic performance of WL and NIR-ICG for identifying pathologic lesions compared to pathology. The statistical significance was set at *p*-values <0.05.

## Results

### General Results

**Figure 2:**
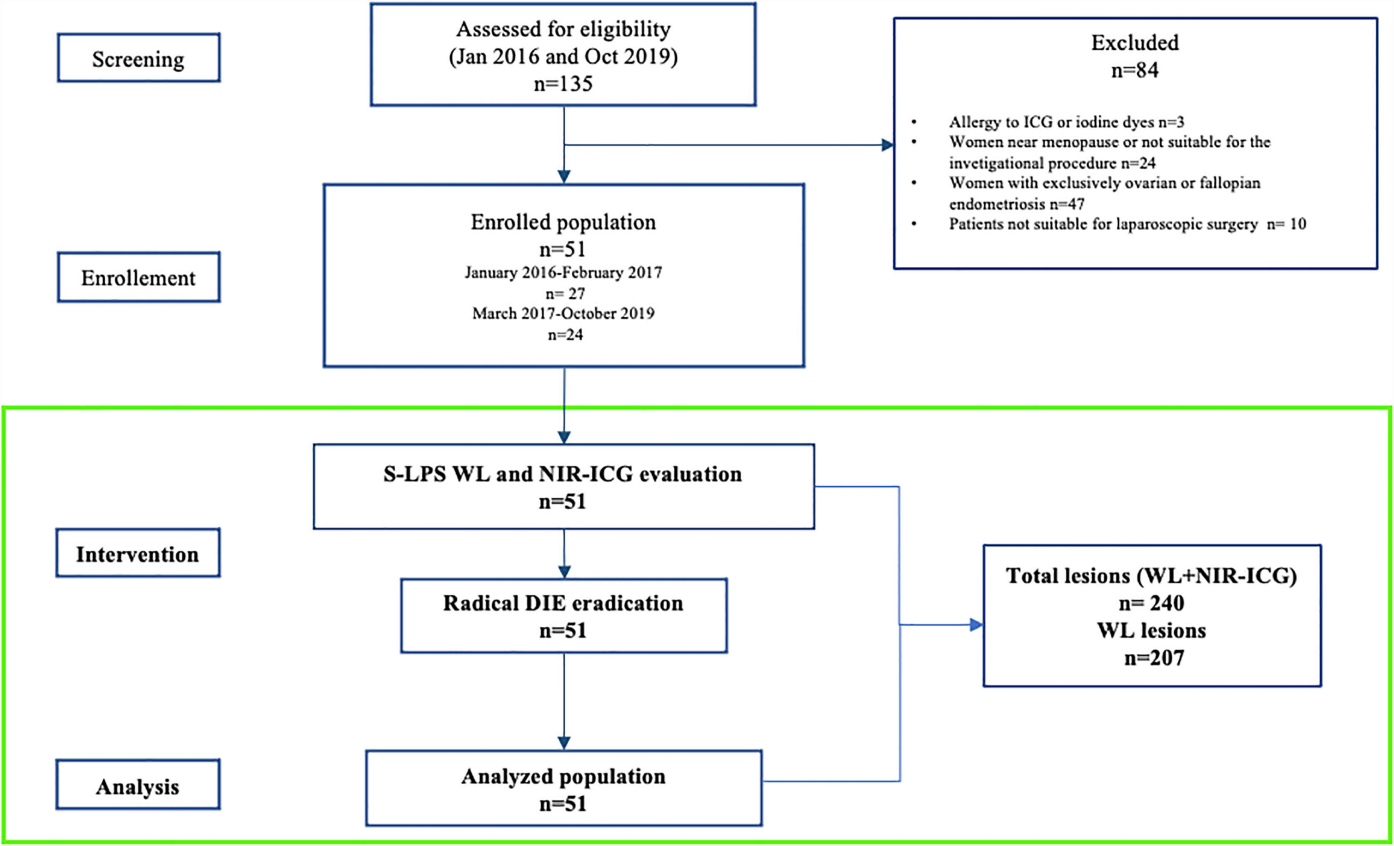
Study flow diagram.

A total of 135 patients with symptomatic endometriosis were screened and 51 were enrolled between January 2016 and October 2019 (**Figure 2**). All patient demographics are listed in **Table 1**. Patients were in premenopausal age between 26 and 47 years old (median age = 35 years) with a median body mass index of 20.5. Nineteen (35%) women had already undergone previous surgery for endometriosis. All patients suffered from severe symptoms referred at the VAS scale. Forty-five patients had an intraoperative assignment to III/IV stage by the rAFS.

**Table 1:** Characteristics of the patients.

Variables	Value	Percentage
All cases	51	100%
Age, years (range)	35 (26–47)	–
Body mass index (range)	20.5 (14–33)	–
ASA class		
1	35	69%
2	16	31%
3	0	–
Previous delivery	18	35%
Prior surgery for endometriosis	19	37%
Preoperative symptoms (VAS)^a^		
Dysmenorrhea	9 (3–10)	86%
Dyschezia	7 (2–10)	59%
Dysuria	7 (4–10)	18%
Dyspaurenia	8 (1–10)	67%
Chronic pelvic pain	6 (2–10)	69%
Stage^b^		
Stage I (minimal)	0	–
Stage II (mild)	6	12%
Stage III (moderate)	19	37%
Stage IV (severe)	26	51%

Data are shown as median/range for the referred positive VAS. Percentage refers to the number of patients according to symptoms.

a Pain is valued with the visual analog scale (VAS) for symptomatic patients.

b According to the rAFS classification.

All patients underwent laparoscopic surgery, and no laparotomy conversions were recorded. The most performed surgery was uterosacral ligament (USL) nodule removal in 80% of the patients, while retrocervical nodule resection was performed in 78% of the cases. Overall, segmental colorectal resection represented 28% of the cases.

**Table 2** reports the surgical procedures performed and the perioperative outcomes observed. There was no increase in operating times because of the use of NIR-ICG imaging because injection of the ICG dye occurred during the preparation of the operating field. ICG median dose injected was 15 mg (range 10.25–24).

**Table 2:** Surgical procedures and perioperative data.

Surgical procedure	Value	Percentage
Ovarian cyst removal	26	51%
Peritoneal removal	36	70%
Retrocervical nodule removal	40	78%
Vaginal nodule removal	14	27%
Uterosacral ligament nodule removal	41	80%
Rectal nodule shaving	12	23%
Segmental resection and anastomosis of sigma-rectum	10	20%
Segmental resection and anastomosis of sigma-rectum plus ileostomy	4	8%
Other procedures (appendectomy, salpingectomy, ureteral stent placement)	14	27%
Operative time (min)^a^	142 (65–375)	–
Dose of ICG injected^a^	15 (10.25–24)	–
Intraoperative complications	0	–
Estimated blood loss (ml)^a^	100 (0–350)	–
Postoperative complications^b^		
Early	9	17.6%
I	2	–
II	5	–
III	2	–
IV	0	–
Late	0	
Hospital stay (no. of days)^a^	2 (1–13)	–

a Data are shown as median/range.

b According to Clavien–Dindo classification.

No hemorrhages, allergic reactions, and/or any type of intraoperative complications were reported. Postoperative complications affected nine (17.6%) patients and were not directly associated to the ICG infusion or to the experimental plan. In particular, only *early* complications were registered: among grade I, one case of urinary retention and one case of fever; among grade II, one case of postoperative bleeding of colorectal anastomosis in postoperative day 2 was noted and controlled by hospitalization and the administration of 500 mg tranexamic acid orally every 8 h for 3 days, three cases of fever needing for antibiotic infusion, and one case of anemia needing red cell-concentrated unit transfusion; among grade III, one case of colorectal anastomosis dehiscence then subjected to resuturing and loop ileostomy creation and one case of vaginal fornix dehiscence after a posterior wall nodule resection needing to be resutured.

### Study Protocol Results

Fifty patients administered with ICG presented tissue fluorescence, except one woman that was completely negative even after a second repeated dose.

**Figure 3:**
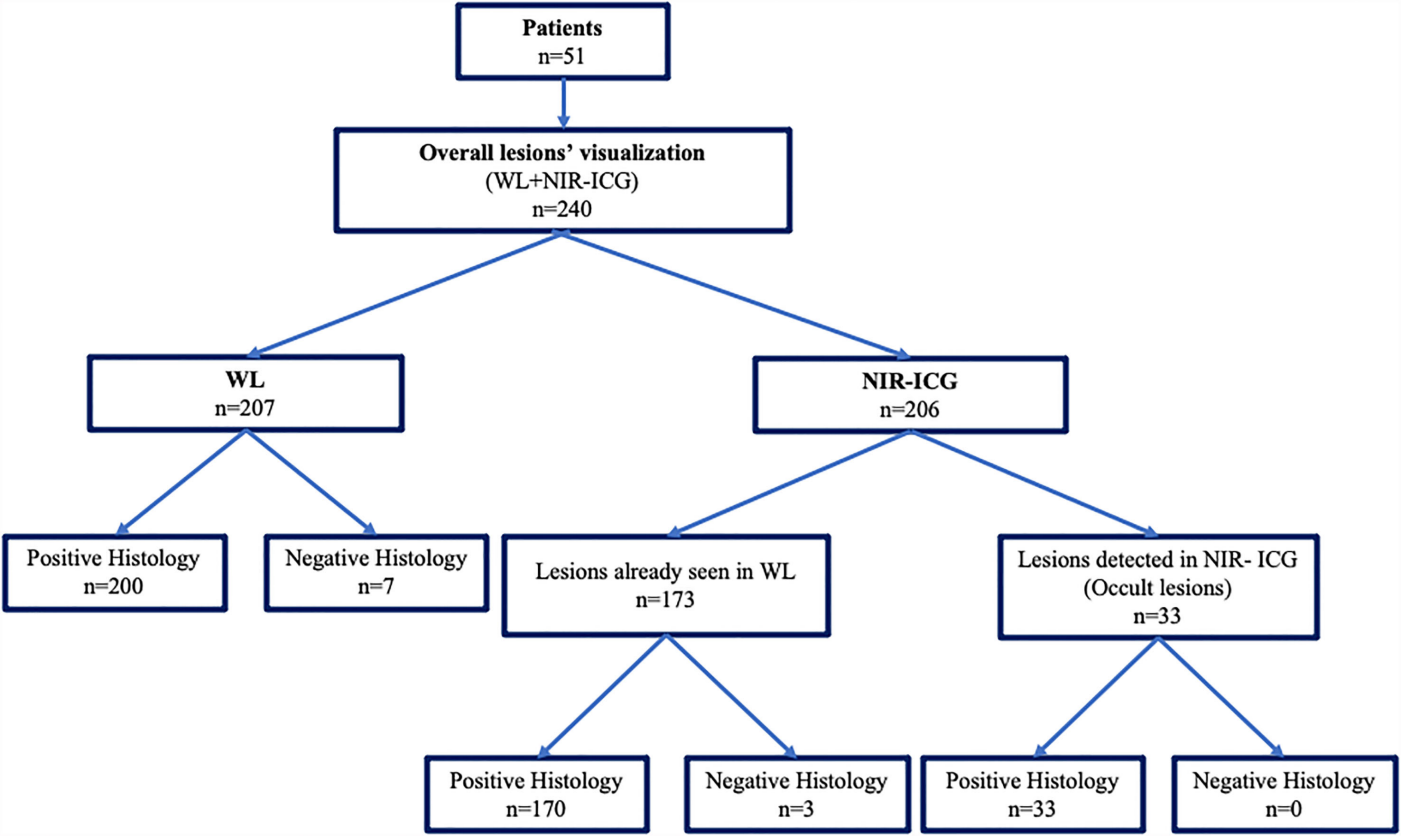
Graphical representation of the collection of intraoperative and pathological data derived from WL vision and the combination of the two techniques (WL plus NIR-ICG).

A total of 240 suspected lesions have been identified with both methods (WL + NIR-ICG). Two hundred and seven (86.2%) lesions out of the overall 240 ones were visualized with WL imaging, and 200 were confirmed to be pathologic (true positive for WL). The remaining 33 out of 240 (13.75%) (false negative for WL) lesions were identified only with NIR-ICG imaging and collected as s-OcL (**Table 3** and **Figure 3**). The s-OcLs were so distributed: 3 (9%) white lesions for PE, while 7 (21%) as retrocervical, 3 (9%) as USL, 11 (33%) as periureteral/ovarian fossa, 4 (12%) as rectal, and 5 (15%) as prevesical/vesical localizations for DIE (**Table 3**). All 33 s-OcLs removed were confirmed to be pathologic (c-OcL = 100%): in particular, 25 (76%) lesions out of 33 harbored occult endometrioses, while 8 (24%) harbored severe sclerosing inflammatory infiltrate. Moreover, 30 (15%) lesions out of the 200 confirmed lesions identified by WL have not visualized by NIR-ICG.

**Table 3:** Intraoperative and pathologic data collection resulting from WL vision and the combination of the two techniques (WL plus NIR-ICG).

Variables	WL visualization	Overall visualization (WL plus NIR-ICG)	Pathology for WL	Overall pathology (WL plus NIR-ICG)	True positive for WL	False positive for WL	True negative for WL^a^	False negative for WL (s-OcL)	c-OcL
Peritoneal endometriosis									
White lesion	21	24	17	20	17	4	21	3	3
Black lesion	16	16	15	15	15	1	16	0	0
Deep infiltrating endometriosis									
Retrocervical nodule	35	42	34	41	34	1	35	7	7
USL nodule	62	65	61	64	61	1	62	3	3
Periureteral/ovarian fossa nodule	20	31	20	31	20	0	20	11	11
Vaginal nodule	11	11	11	11	11	0	11	0	0
Sigma-rectum nodule	26	30	26	30	26	0	26	4	4
Prevesical/vesical nodule	16	21	16	21	16	0	16	5	5
Overall endometriosis									
Total (PE and DIE)	207	240	200	233	200	7	207	33	33

WL, white light visualization mode/expert surgeon eye; c-OcL, confirmed occult endometriosis lesion at WL (=FN); PE, superficial peritoneal endometriosis; DIE, deep infiltrating endometriosis.

a True negative for WL = 51 control biopsies performed in WL.

**Table 4** reports the specific results obtained using NIR-ICG. With NIR-ICG imaging, 206 suspected lesions were identified; 173 of them were already visualized in WL. Two hundred and three suspected lesions out of 206 (98.5%) had pathologic confirmation (true positive for NIR-ICG), while 3 lesions were not confirmed as pathologic (false positive for NIR-ICG). As reported above, 33 lesions further than conventional WL were identified and confirmed at pathology (c-OcL) thanks to NIR-ICG (**Figure 3**).

**Table 4:** Intraoperative and pathologic data collection resulting from NIR-ICG.

Variables	Overall NIR-ICG visualization	NIR-ICG visualization already seen in WL	Pathology for NIR-ICG	True positive for NIR-ICG	False positive for NIR-ICG	True negative for NIR-ICG^a^	False negative for NIR-ICG	c-OcL
Peritoneal endometriosis								
White lesion	14	11	13	13	1	14	7	7
Black lesion	9	9	9	9	0	9	6	6
Deep infiltrating endometriosis								
Retrocervical nodule	41	34	40	40	1	40	1	1
USL nodule	61	58	57	57	1	58	4	4
Periureteral/ovarian fossa nodule	29	18	29	29	0	29	2	2
Vaginal nodule	10	10	10	10	0	10	1	1
Sigma-rectum nodule	27	23	27	27	0	27	3	3
Prevesical/vesical nodule	15	10	15	15	0	15	6	6
Overall endometriosis								
Total (PE and DIE)	206	173	203	203	3	206	30	33

NIR-ICG, near-infrared visualization mode with indocyanine green; c-OcL, confirmed occult lesion at NIR-IGC (=FN at WL); PE, superficial peritoneal endometriosis; DIE, deep infiltrating endometriosis.

a Number of control biopsies performed in NIR-ICG + TN of PE and DIE.

Regarding WL vision, the overall PPV and NPV were 96.6% and 86.3%, while Se and Sp were 85.8% and 96.7% (**Table 5**). NIR-ICG vision showed PPV of 98.5%, NPV of 87.1%, Se of 87%, and Sp of 98.5% (**Table 5**), confirming that this kind of imaging is an excellent diagnostic and screening test (*p* = 0.001 and *p* = 0.835, according to McNemar’s and Cohen’s kappa tests, respectively).

**Table 5:** Comparison between the NIR-ICG and WL for each surgical site in the whole population.

Variable	Vision	PPV (%)	NPV (%)	Sensitivity (%)	Specificity (%)	Accuracy (%)	McNemar’s test	Cohen’s kappa
Peritoneal endometriosis								
White lesion	WL	81.0	87.5	85.0	84.0	46.7	0.301	0.667
	NIR-ICG	92.9	66.7	65.0	93.3	40.0		
Black lesion	WL	93.8	100	100	94.1	50.0	0.125	0.751
	NIR-ICG	100	60.0	60.0	100	60.0		
Deep infiltrating endometriosis								
Retrocervical nodule	WL	97.1	83.3	82.9	97.2	45.4	0.109	0.874
	NIR-ICG	97.6	97.6	97.6	97.6	50.0		
USL nodule	WL	98.4	95.4	95.3	98.4	48.8	0.179	0.927
	NIR-ICG	98.3	93.5	93.4	98.3	48.3		
Periureteral/ovarian fossa nodule	WL	100	64.5	64.5	100	39.2	**0.002**	0.768
	NIR-ICG	100	93.5	93.5	100	48.3		
Vaginal nodule	WL	100	100	100	100	50.0	1.0	0.960
	NIR-ICG	100	90.9	90.9	100	47.6		
Sigma-rectum nodule	WL	100	86.7	86.7	100	46.4	**0.01**	0.876
	NIR-ICG	100	90.0	90.0	100	47.3		
Prevesical/vesical nodule	WL	100	76.2	76.2	100	43.2	**0.001**	0.705
	NIR-ICG	100	71.4	71.4	100	41.6		
Overall endometriosis								
Total (PE and DIE)	WL	96.6	86.3	85.8	96.7	46.3	**0.001**	0.835
	NIR-ICG	98.5	87.1	87.0	98.5	46.5		

NIR-ICG, near-infrared visualization vision with indocyanine green; WL, white light vision; PPV, positive predictive value; NPV, negative predictive value; PE, superficial peritoneal endometriosis; DIE, deep infiltrating endometriosis. The bold style means that the values reported are statistic significant.

As far as PE is concerned, NIR-ICG demonstrated higher values of PPV and specificity than WL, while NPV and sensitivity were lower. The accuracy of the NIR-ICG was lower than WL regarding white lesion (40% *vs*. 46.7%); conversely, it was superior in recognizing black lesion (60% *vs*. 50%).

As far as DIE is concerned, the two visualization approaches demonstrated superimposable values of Sp, while Se and accuracy resulted higher for NIR-ICG for the visualization of periureteral/ovarian fossas and colorectal nodules (McNemar’s test: *p* = 0.002 and 0.768 and Cohen’s kappa test: *p* = 0.01 and 0.876). Conversely, WL demonstrated superiority than NIR-ICG in the recognition of prevesical/vesical lesions (McNemar’s test: *p* = 0.001, Cohen’s kappa test: *p* = 0.705).

The overall accuracy of both methods was 46% with McNemar’s (*p* = 0.001) and Cohen’s kappa tests (*p* = 0.83), revealing that both methods, regardless of the operator, should always be integrated to ensure complete eradication of endometriotic lesions (**Table 5**).

**Figure 4:**
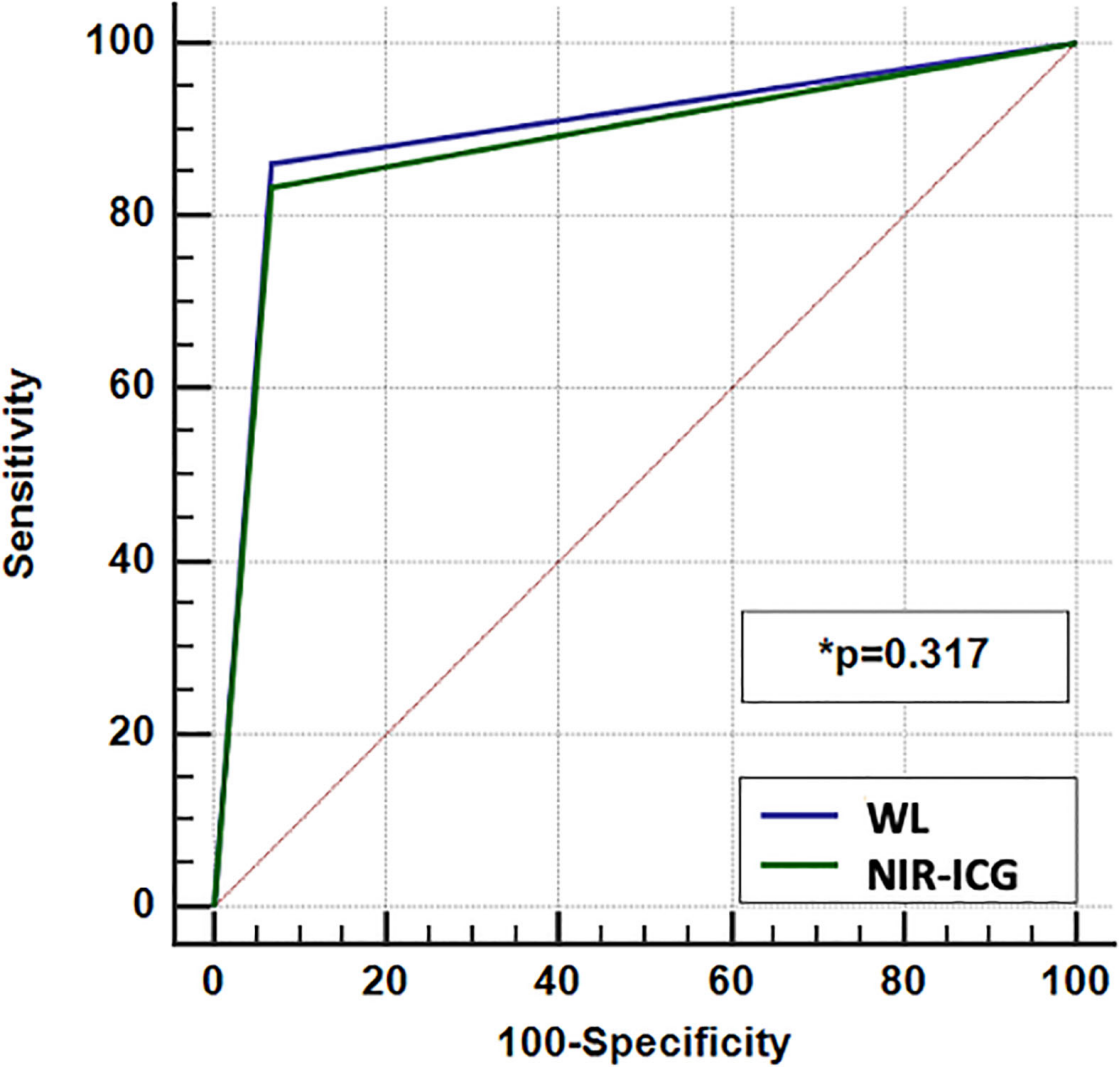
ROC curves of the two approaches (white light and near-infrared indocyanine green).

**Figure 4** details the ROC curves of the two approaches. The areas under the curves (AUCs) were >0.8, and the diagnostic powers of the two methods were not statistically different (*p* = 0.31).

## Discussion

### Results in the Context of Published Literature

The role of surgery in endometriosis is to remove the affected tissues to obtain pain relief, to improve fertility, and to abate not only the persistence of disease but also the risk of recurrence that occurs from 20% up to 50% of women at 2 and 5 years after treatment (9, 32, 33).

On the other hand, endometriosis is not cancer, so the excessive search for surgical radicality can imply an increase in perioperative morbidity and can cause serious functional damage, especially if the resection of tissues affected by endometriosis is associated with the removal of healthy tissues surrounding the diseased or mimicking them (10, 34–43).

Furthermore, the sensory perception of the surgeon, albeit an expert one, in identifying intraoperatively suspect lesions for endometriosis based on location, color, size, and depth has obtained well-known limits. Indeed, Stegmann et al. found that the PPV of using only the impression of an experienced surgeon to identify histologically positive lesions was 64.0%, and the NPV was 88%, while the Se and Sp of using this method were 98% and 21%, respectively (34).

Furthermore, the possibility of not recognizing outbreaks of endometriosis that is not visible because it is microscopic or hidden (occult endometriosis) in 6%–13% of the cases may further worsen the effectiveness of surgical clearance (6, 9, 44).

For these reasons, different approaches to increase the potential for intraoperative recognition of endometriosis (enhanced vision) have been investigated using different dyes or technologies such as 3D robotic vision, with different efficacy and safety profiles (9–11, 21–24, 45–47). The results of the Gre-Endo trial seem to answer to the need of an “enhanced vision” and the utility of an intraoperative screening test.

Moreover, in association with the intraoperative diagnostic role of NIR-ICG, a challenging additional advantage seems to be the capability of distinguishing the diseased tissue from the surrounding healthy ones and to evaluate the residual vascularization of noble organs subjected to dissection and/or eradicating surgery such as the rectum and ureter (10, 11, 48, 49).

In this study, we confirmed what was already found in recent literature about the identification of lesion and the definition of the extent and limitation of lesions from healthy tissues (9–11, 21–25, 45, 48, 49). In addition, we defined the diagnostic power of the method, which was not systematically investigated before.

In this cohort, NIR-ICG showed higher values of Se and Sp compared with the standard WL (87% *vs*. 85.8% and 98.5% *vs*. 96.7%, respectively). Moreover, the overall values registered at McNemar’s (*p* = 0.001) and Cohen’s kappa (*p* = 0.83) tests showed that NIR-ICG is an excellent screening and diagnostic confirmation test compared with WL. Cohen’s kappa is higher than 0.4 for all types of lesion observed: it means that the evaluation of the two tests is good and independent from the observer.

In McNemar’s test, on the other hand, we noticed the statistical significance in the overall results, in the USL lesions, and on the rectum and bladder localization: it means that NIR-ICG and WL should always be integrated to provide the most complete eradication of endometriosis, in particular in the sites described above.

Finally, the AUC of ROC curves for WL and NIR-ICG resulted in values >0.8 that in association with the K Cohen results >0.4 encountered, represents a positive evaluation of the diagnostic tests with excellent diagnostic powers.

Subsequently, we could sustain that the two approaches should be used sequentially during the same surgery to compare the lack and the gains of one against the other.

Moreover, in this series, the diagnostic power of NIR-ICG imaging seems also maintained in two situations of vascularization impairment, i.e., i) in patients with previous surgery and ii) after surgical dissection and tissue cruentation (data not shown). However, considering the few cases analyzed, further prospective studies are needed to confirm the excellent result for this subset of patients.

Finally, it is necessary to underline, thanks to NIR-ICG, that 33 lesions (c-OcL), which otherwise would not have been removed with standard approaches, had been resected, with a gain of 16.5% in the count of lesions removed only by WL and a gain of 14.16% in the overall count of lesions resected by the combination of the methods.

Interestingly, no iatrogenic opening of the bowel during conservative eradication procedures nor intraoperative ureteral lesions or postoperative fistulas occurred; moreover, the postoperative urinary retention observed was only 2%, lower than the rate reported in the literature (36). These interesting low rates of perioperative complications may be possible, thanks to the already noticed benefits of fluorescence-guided surgery (10, 48–50). Only one case of anastomotic colorectal dehiscence occurred, but the anastomotic vascularization (48) was not investigated because of the washout of ICG employed at the beginning of the surgery for the study purpose. Further studies focused on complication rate with adequate population are needed to confirm these results.

To our knowledge, this is the first prospective trial with the most consistent population subjected to the same homogeneous procedure (surgery, ICG dose of injection, time of observation, dedicated pathologist) (51).

Recently, Siegenthaler et al. (11) found that NIR-ICG resulted as useful for identifying the extent of lesions, allowing for the resection of nodules and the preservation of healthy tissue surrounding the diseased, but the results were not satisfactory regarding detection rate. In fact, in this study, the PPV reported for WL, NIR-ICG, and the combination of the two methods were 89.8%, 68.8%, and 86.7%, respectively, while in our cohort, the values noticed were 96.6%, 98.5%, and 97.6%, respectively. Moreover, s-OcLs for NIR-ICG in Siegenthaler et al. (11) were at least 22%, but only one lesion was a c-OCL (4.5%). Those results may differ from our results for several factors, identifiable in the different selection of population for the rAFS stage: different times of intraoperative observation for a subset of the population [only 35 (55.6%) patients of that cohort received a comparable observation time with the Gre-Endo trial] and different doses of ICG adopted. Moreover, another difference was the fact that we considered as pathologic not only endometriosis per se but also lesions characterized by acute or chronic sclerosing inflammatory infiltrate (28, 29, 51).

### Strengths and Limitations of the Study

The strengths of this study are the single-center prospective design, the considerable population enrolled subjected to the same experimental procedure and the standardized surgery by a limited team of high-volume surgeons, and a dedicated pathologist (51).

The limitations of the present study include the exclusion by the investigation of the adnexal endometriosis and the higher percentage (~89%) of advanced endometriosis stage (stages III and IV for rAFS) that could represent a bias of selection.

However, this type of population enrolment could be explained by the fact that our hospital is a third referral center to triage treatment of women with endometriosis who may be suffering from an advanced stage that cannot be treated elsewhere. Moreover, according to recent literature, the enrolment of higher stage of disease may have worsened the detection rate results of the present study (11). These limitations could be partially solved by the fact that in our institutions, surgical care and laparoscopic evaluation are standardized and there is the ability to partially overcome differences such as preoperative patient selection, surgical strategies, and intraoperative visualization.

### Future Perspectives

This type of approach has recently been associated with a promising improvement in the quality of life (25), but further studies are needed to establish the real benefit in terms of pain relief, recurrence rate, and fertility rate resulting from the strengthening of the eradication power found. In addition, another direction in which research should be directed should be its use in a population that includes all stages of disease and, therefore, also focuses on the lower stages of disease.

### Conclusions

The use of NIR-ICG alone and above all combined with WL during laparoscopy for endometriosis showed good results in intraoperative detection rate and fluorescence-guided surgery. Furthermore, NIR-ICG allowed surgeons to remove occult lesions that otherwise would remain, leading to possible greater postoperative pain and a higher risk of persistence and relapse.

Further prospective studies that overcome the possible biases of this study are warranted to validate and confirm these results and permit a diffusion of fluorescence-guided endometriosis surgery as a useful aid for a more effective and safe surgery.

## Data Availability Statement

The raw data supporting the conclusions of this article will be made available by the authors, without undue reservation.

## Ethics Statement

The studies involving human participants were reviewed and approved by Ethics Committee of Fondazione Policlinico Universitario “A. Gemelli”—IRCCS, Rome, Italy (Prot.sf. A.287/C.E./2013). The patients/participants provided their written informed consent to participate in this study.

## Author Contributions

Study concepts: LT, VV, GV, and FC. Study design: LT, GV, and GF. Data acquisition: VV, LT, and MN. Quality control of data and algorithms: GS, FC, and SG. Data analysis and interpretation: GV, LT, GF, and LP. Statistical analysis: GV, LP, and GF. Manuscript preparation: LCT, GV, and SG. Manuscript editing: LT, VV, GS, and GV. Manuscript review: all authors. All authors contributed to the article and approved the submitted version.

## Conflict of Interest

The authors declare that the research was conducted in the absence of any commercial or financial relationships that could be construed as a potential conflict of interest.

## Publisher’s Note

All claims expressed in this article are solely those of the authors and do not necessarily represent those of their affiliated organizations, or those of the publisher, the editors and the reviewers. Any product that may be evaluated in this article, or claim that may be made by its manufacturer, is not guaranteed or endorsed by the publisher.
